# Comparative physiological and transcriptomic analysis reveal *MdWRKY75* associated with sucrose accumulation in postharvest ‘Honeycrisp’ apples with bitter pit

**DOI:** 10.1186/s12870-022-03453-8

**Published:** 2022-02-17

**Authors:** Chen Sun, Weiwei Zhang, Haiyong Qu, Longfei Yan, Lixia Li, Yuqi Zhao, Hongqiang Yang, Hua Zhang, Gaifang Yao, Kangdi Hu

**Affiliations:** 1 School of Food and Biological Engineering, Hefei University of Technology, Hefei, 230009 China; 2 College of Horticulture Science and Engineering, State Key Laboratory of Crop Biology, Shandong Agricultural University, Tai’an, 271001 China; 3 College of Horticulture, Qingdao Agricultural University, Qingdao, 266109 China

**Keywords:** Apple (*Malus domestica*), Calcium deficiency, Sucrose-metabolism, *MdWRKY75*, *MdSWEET1*

## Abstract

**Background:**

Calcium (Ca) deficiency can cause apple bitter pit, reduce the quality and shelf life. WRKY transcription factors play essential role in plant response to multiple disorders. However, the underlying mechanisms causing bitter pit in apple fruit due to Ca deficiency during storage is extremely limited.

**Results:**

In the present study, the nutritional metabolites and reactive oxygen species (ROS) were compared in Ca-deficient and healthy apple fruit (CK) during storage. Results showed that Ca-deficient apples sustained significantly higher production of ROS, PPO activity, flavonoids, total phenol, total soluble solids (TSS), and sucrose contents, but the contents of Ca, H_2_O_2_, titratable acids (TA), glucose and fructose were significantly lower than those of CK during storage. Principal component analysis (PCA) showed that TSS, •O_2_^−^, PPO, malondialdehyde (MDA) and Ca were the main factors, and TSS had a positive correlation with sucrose. Furthermore, transcriptome analysis revealed that WRKYs were co-expressed with sucrose metabolism-related enzymes (*SWEETs*, *SS*, *SPS*). qRT-PCR and correlation analysis indicated that *MdWRKY75* was correlated positively with *MdSWEET1*. Moreover, transient overexpression of *MdWRKY75* could significantly increase the sucrose content and promote the expression of *MdSWEET1* in apple fruit.

**Conclusions:**

Calcium deficiency could decrease antioxidant capacity, accelerate nutritional metabolism and up-regulate the expression of WRKYs in apple with bitter pit. Overexpression of *MdWRKY75* significantly increased sucrose accumulation and the expression of *MdSWEET1*. These findings further strengthened knowledge of the basic molecular mechanisms in calcium deficiency apple flesh and contributed to improving the nutritional quality of apple fruit.

**Supplementary Information:**

The online version contains supplementary material available at 10.1186/s12870-022-03453-8.

## Background

Apples with high nutritional value are favored by consumers. However, the physiological obstacles represented by bitter pit result in serious economic losses for postharvest apples [1]. Bitter pit is a common physiological disorder caused by calcium deficiency in the tree during fruit development and storage of apples, and symptoms frequently appear during or after cold storage; in severe cases, they may appear before harvest [2–4]. In fruit, approximately 40% of free calcium is located in the vacuole, and the other 60% is located in the cell wall. High calcium content is an important factor regulating cell homeostasis. Normal cell metabolism requires the cell membrane to directly contact free calcium to maintain proper cell membrane stability and function [5]. Metabolic changes of Ca^2+^ content in these compartments might be related to Ca^2+^ depletion in apoplastic solution, which can weaken plasma membrane structures, leading to cell death and bitter pit symptoms [6, 7].

As essential nutrients for plants, Ca^2+^ play an important role in regulating fruit development and ripening [8]. Calcium pectinate can affect the sensitivity of plants to fungal infection and participate in the regulation of the ripening process of fruit [9]. Reactive oxygen species (ROS), including ·O_2_^−^ and H_2_O_2_, which are highly reactive and toxic, and can lead to the oxidative destruction of cells, are usually used as indicators to measure the senescence of fruits. And malondialdehyde (MDA) is an important product of membrane lipid oxidation [10]. It was reported that exogenous calcium ions delayed fruit ripening and maintained nutritional quality and appearance. With an increase in the calcium concentration, papaya fruit ripening and the senescence process were inhibited, slowing down softening and prolonging storage life [11, 12]; firmness and TSS increased and postharvest decay was reduced in strawberries; total phenolics and total antioxidant capacity increased, and TA, ascorbic acid and decay rate decreased in sweet cherry [13]; protein, ascorbic acid, TA and carbohydrates decreased in banana [14]; and calcium spray decreased ascorbic acid and sugar content and stimulated catalase enzyme activity and pathogen defense genes during storage in grape berries [15, 16]. In apples, low levels of calcium cause faster ripening, and ascorbic acid and firmness are lower [17]. Furthermore, spraying calcium chloride on preharvest apples can effectively reduce the occurrence of bitter pit, and it can significantly improve the firmness and appearance of apple to enhance shelf life [18–20]. Calcium can reduce fruit cracking and promote the healing of mechanical injury [21]. Thus, calcium deficiency can cause many disorders that influence the edible quality and postharvest storage of fruit.

Sugar content is an important determinant of fruit edible quality, and bitter pit increase TSS and sugar contents, such as sucrose content [7]. Sucrose synthesis (SS) can reversibly catalyze sucrose to fructose, glucose and uridine diphosphate glucose (UDP-glucose) [22]. Sucrose-phosphate synthase (SPS) is an important enzyme in the irreversible reaction that catalyzes UDPG and 6-phosphate-fructose to sucrose [23]. In peaches, nitric oxide (NO) enhances gene expression and the activities of SPS and SS and leads to an increase of sucrose content [24]. In apple fruit, it was reported that sodium nitroprusside (SNP) treatment delayed loss of quality by enhancing *MdSPS* and *MdSS* expression and then increasing the sucrose content [25], and the sucrose transporter *MdSUT4.1* participates in the regulation of fruit sugar accumulation [26]. SWEETs were identified as sugar transporters responsible for fruit sugar accumulation, and *MdSWEET9b* and *MdSWEER15a* were involved in regulating fruit sugar accumulation in apple [27]. In pear fruit, *PuWRKY31* can accelerate the synthesis of sucrose by binding to *PuSWEET15* [28]. However, whether WRKY TFs regulate the sugar transporter *SWEETs* in calcium deficiency apple fruit remains unclear.

To explore the molecular mechanisms of postharvest quality in apple fruit calcium deficiency caused by bitter pit, the content of nutrients and antioxidant capacity in apple fruit during storage period were determined. The main factors involved in bitter pit (Ca, TSS, TA and MDA) were screened by PCA and correlation analysis, and the sugar transporter-related enzyme *MdSWEET1* and *MdWRKY* TFs were identified by bioinformatics and qRT-PCR analysis. Furthermore, *MdWRKY75* was transformed and identified by apple transient expression system. These results will provide new insights into candidate genes for sugar accumulation in calcium deficiency apples and the improvement of fruit quality.

## Results

### Low calcium content caused bitter pit disorder, which shortened the shelf life of apple fruit

**Fig. 1:**
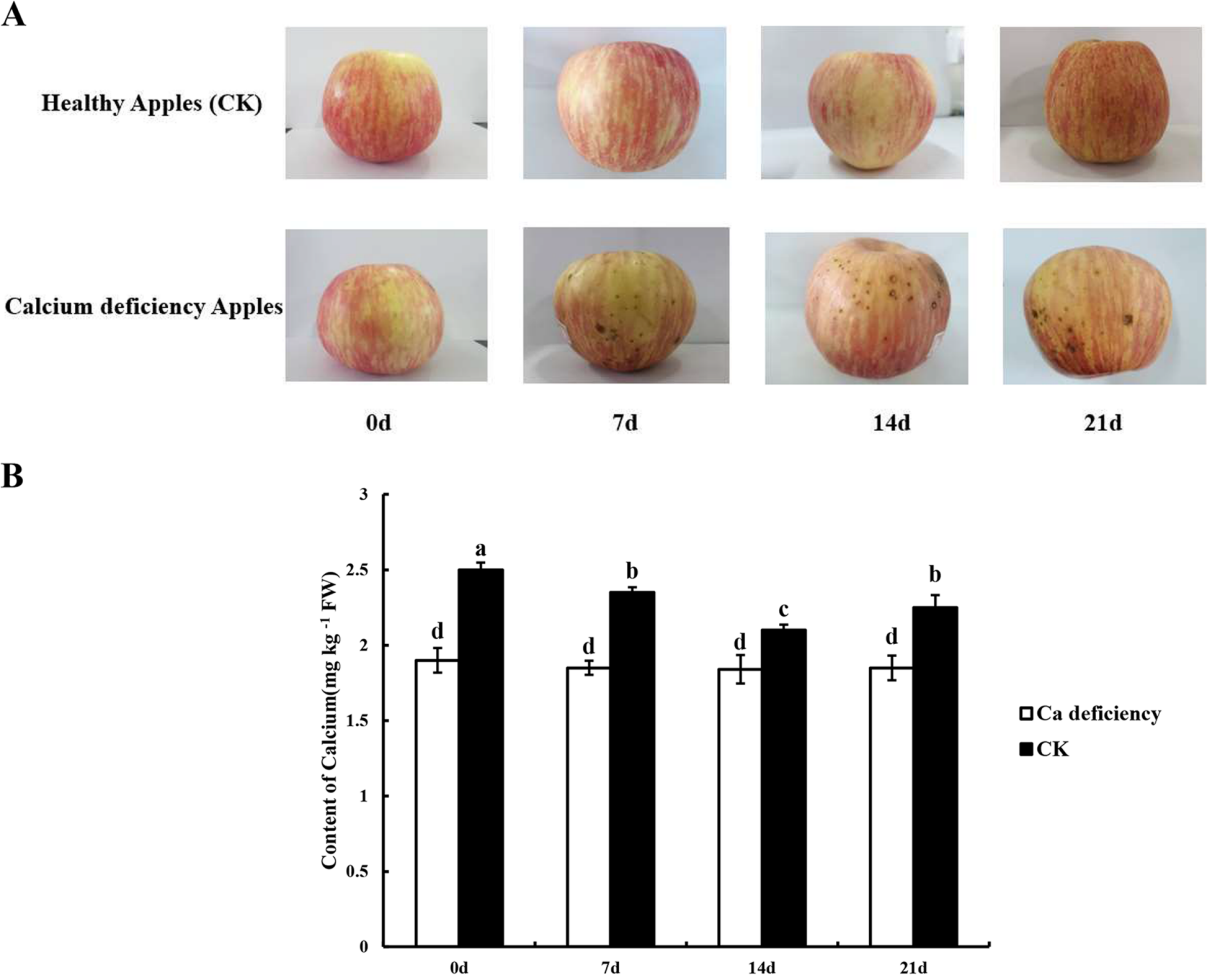
Changes of the phenotype and Ca contents in postharvest calcium deficiency and healthy apples. **A** Changes of the phenotype in the calcium deficiency and healthy apples at 0, 7, 14 and 21 DAS. **B** Changes of Ca contents in postharvest calcium deficiency and healthy apples at 0, 7, 14 and 21 DAS. Different lower-case letters above the columns stand for significant difference between two values (*p* < 0.05) at the same time point

In our experiments, calcium deficiency and healthy apple fruit (CK) were analyzed during the storage period. As shown in Fig. 1A, calcium deficiency apple fruit exhibited bitter pit disorder 7 days after storage (DAS), and severity was increased thereafter, while CK did not show disorder characteristics during the storage period. The apple surfaces of bitter pit disorder turned dark yellow compared to CK at 21 DAS. Furthermore, the calcium content of apple fruit was determined, and the results showed that the calcium content in calcium deficiency apple fruit was significantly lower than that of the CK fruit during the storage period (*p* < *0.05*) (Fig. 1B). This showed the reliability of bitter pit disorder in calcium deficiency apples.

### Comparison of H_2_O_2_, •O_2_^–^, MDA and activity of PPO in calcium deficiency and CK apple fruit during the storage period

**Fig. 2:**
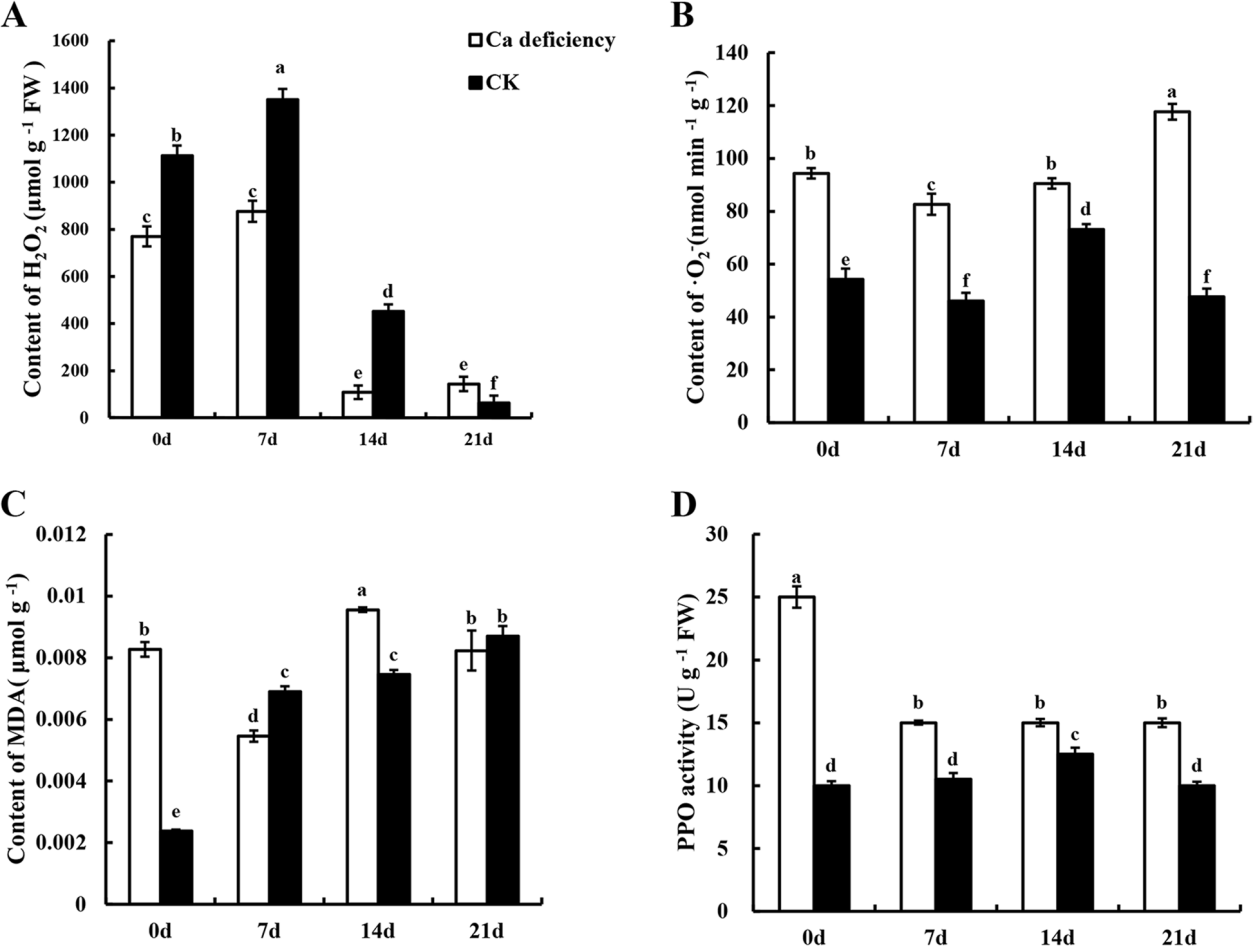
The changes of reactive oxygen species (ROS) in postharvest healthy apples and calcium deficiency apples. **A** hydrogen peroxide, **B** superoxide anion, **C** malondialdehyde and **D** the activity of polyphenol oxidase. Data are presented as means ± SD (*n* = 3). Different lower-case letters above the columns stand for significant difference between two values (*p* < 0.05) at the same time point

The results showed that calcium deficiency apple fruit maintained lower levels of H_2_O_2_ during postharvest storage. Significantly lower levels of H_2_O_2_ were observed in calcium deficiency fruit than in the CK fruit during the storage period until 14 DAS (*p* < *0.05*) (Fig. 2A). The H_2_O_2_ content of the control fruit and calcium deficiency fruit decreased from 7 to 14 DAS. At 14 DAS, the H_2_O_2_ content of calcium deficiency fruit was nearly five fold lower than that in CK. Thereafter, the H_2_O_2_ content in the control fruit decreased gradually due to senescence and rot with prolonged storage time. The production of H_2_O_2_ in calcium deficiency fruit was accelerated from 0 to 7 DAS and reduced rapidly thereafter.

As shown in Fig. 2B, the rate of •O_2_^–^ production remained high during storage irrespective of disorder. Compared with the control fruit, significantly higher •O_2_^−^ production was observed in calcium deficiency apple fruit during the entire storage period (*p* < *0.05*).

Figure 2C showed that the MDA content in calcium deficiency fruit was decreased at first, then enhanced rapidly during the entire storage period. At 7 DAS, the levels of MDA in calcium deficiency apple fruit were significantly lower than those in CK. At 14 DAS, the levels of MDA in calcium deficiency apple fruit were higher than those in the control. Finally, at 21 DAS, the MDA content was approximately the same regardless of disorder.

Figure 2D showed the change in polyphenol oxidase (PPO) activity in apple fruit throughout storage. At 0 DAS, the activity of PPO in calcium deficiency apple fruit was nearly 2.5-fold higher than that in CK. Then, the activity of PPO in calcium deficiency apple fruit decreased rapidly and was maintained at a stable state but was always higher than that in control apple fruit during the entire storage time (*p* < *0.05*). The activity of PPO in control apple fruit rose slowly at first and declined slightly from 14 to 21 DAS.

### Comparison of flavonoids and total phenols in calcium deficiency apple fruit with CK fruit during the storage period

**Fig. 3:**
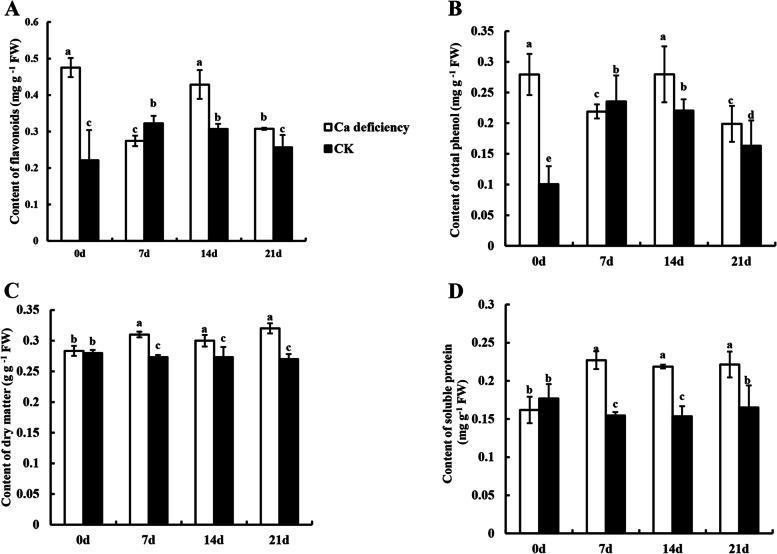
The changes of nutrients in postharvest healthy apples and calcium deficiency apples. **A** flavonoids, **B** total phenols, **C** dry matter and **D** soluble protein. Data are presented as means ± SD (*n* = 3). Different lower-case letters above the columns stand for significant difference between two values (*p* < 0.05) at the same time point

To better understand the improved appearance quality in calcium deficiency apple fruit relative to CK, we determined the contents of flavonoids and total phenols. Figure 3A showed that the flavonoid contents in calcium deficiency apple fruit were dropped at 7 DAS, then increased at 14 DAS and reduced thereafter. The trend of flavonoids in control apple fruit was similar to that of calcium deficiency apple fruit except at 0 DAS. Until 21 DAS, the flavonoid contents tended to be consistent between calcium deficiency apples and the control apple fruit (*p *< 0.05).

In Fig. 3B, there was a significant difference in total phenols between the control apples and calcium deficiency apples at 0 DAS. The total phenols in calcium deficiency apples decreased at 7 DAS, then increased at 14 DAS and reached the lowest value at 21 DAS. The total phenols of the control apple fruit increased from 7 to 14 DAS and dropped at 21 DAS. Similar to the change trend of flavonoid content, the total phenols in calcium deficiency apple fruit were always higher than those of the control apple fruit (except at 7 DAS)(*p *< 0.05).

### Analysis of dry matter and soluble protein content in calcium deficiency apple fruit

It was showed that dry matter and soluble protein increased in calcium deficiency apple fruit. During calcium deficiency apple fruit storage for 21 days, the dry matter content increased slightly and was always higher than that of the control apple fruit (*p* < *0.05*). However, dry matter in control apple fruit did not obviously change during the entire storage period (Fig. 3C). Similarly, the change trend of soluble protein content was the same trend as dry matter content. During 0 to 7 DAS, the content of soluble protein increased rapidly and then maintained at a high level (Fig. 3D). In contrast, it was reduced slightly in control apple fruit at the beginning of storage, and then there was a slight increase within 14 to 21 DAS. The content of soluble protein in control apple fruit was always lower than that in calcium deficiency apple fruit (*p* < *0.05*).

### Identification of TA, TSS, ascorbic acid, ratio of TSS/TA and soluble sugars in calcium deficiency apple fruit

**Fig. 4:**
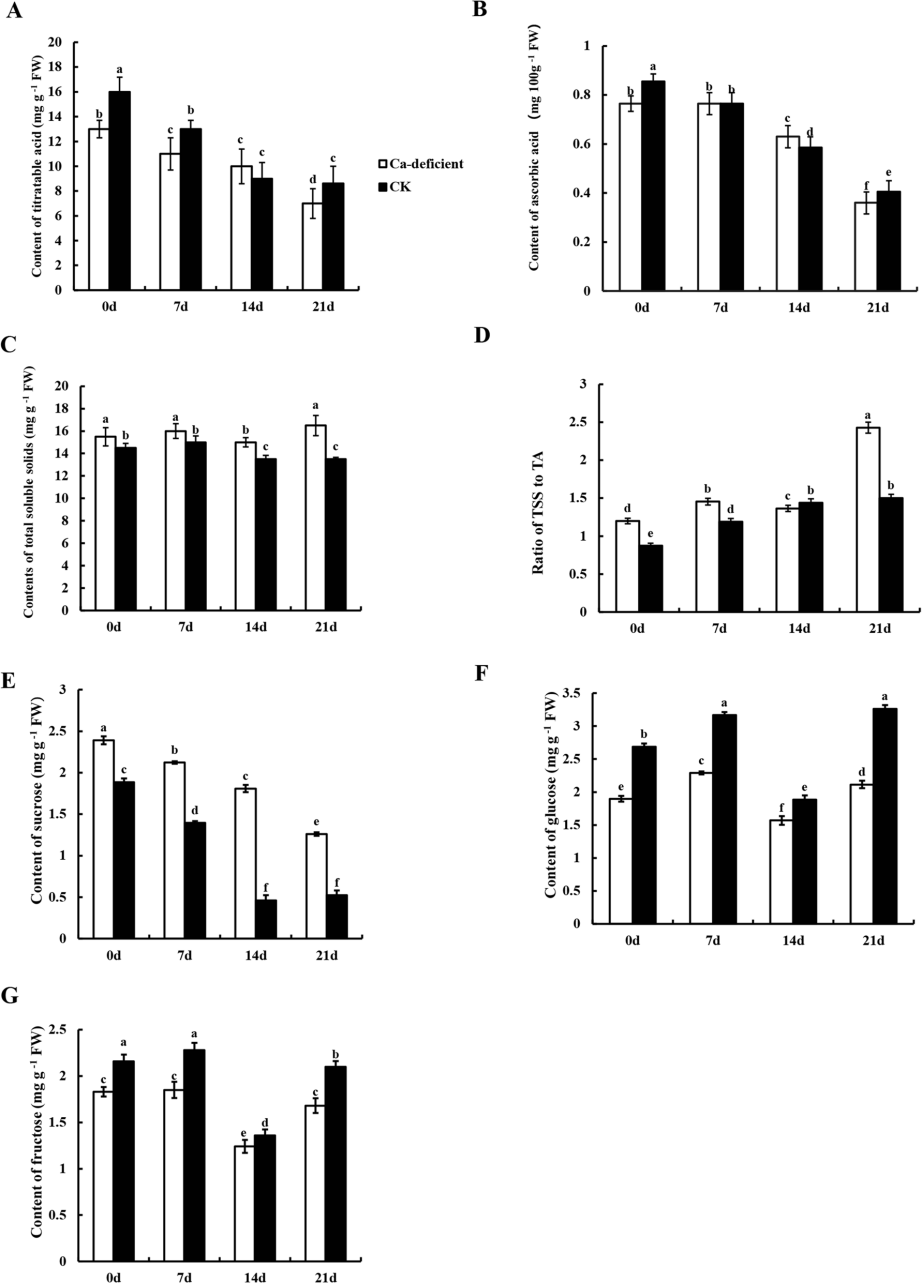
The changes of sugar acid substance in postharvest healthy apples and calcium deficiency apples. **A** titratable acid, **B** ascorbate, **C** total soluble solids, **D** ratio of TSS to TA, **E** sucrose**, F** glucose and **G** fructose. Data are presented as means ± SD (*n* = 3). Different lower-case letters above the columns stand for significant difference between two values (*p* < 0.05) at the same time point

Generally, most of the total soluble substances are soluble sugars, and their contents can directly reflect the ripeness and quality of the fruit. Figure 4 shows the changes in TA, ascorbate, TSS, ratio of TSS/TA and soluble sugars in calcium deficiency apple fruit and control apple fruit. During the whole storage period, TA showed a downward trend in apples regardless of calcium deficiency (Fig. 4A). The TA content in control apple fruit was always higher than that in calcium deficiency apple fruit (*p* < 0.05).

The content of ascorbic acid always decreased during the full storage period in the calcium deficiency and the control apple fruit, and it was the lowest at 21 DAS (Fig. 4B). There was no significant difference in ascorbic acid between the calcium deficiency and control apple fruit at mid-storage. However, at the beginning and late stage of storage, ascorbic acid in control apple fruit was higher than that in calcium deficiency apple fruit (*p *< 0.05).

The change of TSS is shown in Fig. 4C. During the entire storage time, TSS increased slightly in calcium deficiency apple fruit and was always higher than that in control apple fruit (*p* < 0.05). This means that calcium deficiency apple fruit sugar accumulates faster than that in control apple fruit.

The ratio of TSS/TA is an important index for evaluating the flavor of apples. During storage time, the ratio of TSS/TA in calcium deficiency apples was always higher than that of control apples (Fig. 4D). In particular, the ratio of TSS/TA in calcium deficiency apple fruit was significantly higher than that in control apple fruit (except at 14 DAS) (*p* < *0.05*).

During storage time, the sucrose content presented a declining trend in apple fruit, and it was always significantly higher in calcium deficiency apple fruit than in control apple fruit (*p* < *0.05*) (Fig. 4E).

The glucose contents in calcium deficiency and control fruit shared the same trends. The glucose contents increased from 0 to 7 DAS, decreased from 7 to 14 DAS, and finally increased from 14 to 21 DAS (Fig. 4F). During the whole storage time, the glucose contents of calcium deficiency apple fruit were always significantly lower than those of control apple fruit (*p* < *0.05*).

During storage, the fructose content was always lower in calcium deficiency apple fruit than in the control fruit (Fig. 4G). At 14 DAS, the fructose contents of calcium deficiency apple fruit and control apple fruit tended to be consistent. However, the fructose content of the control apple fruit was significantly higher than that of calcium deficiency apple fruit (except at 14 DAS) *(p* < *0.05)*.

### PCA and correlation analysis of the changes in bioactive substances in apple fruit

**Fig. 5:**
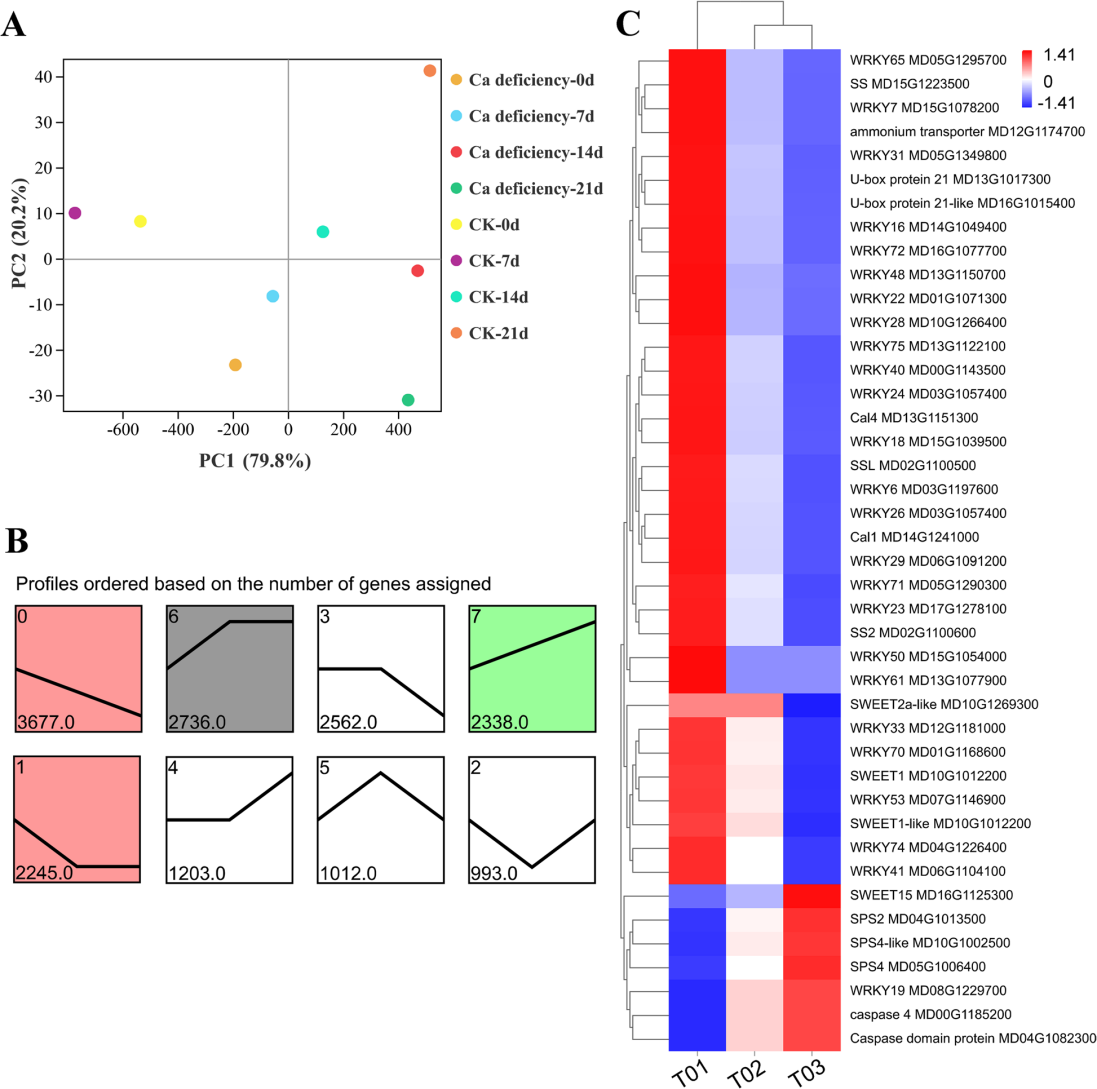
Principal component analysis (PCA) and screening differentially expressed genes (DEGs) from transcriptomic analysis. **A** The main factors of the postharvest calcium deficiency and CK apple fruit was determined during the storage periods by PCA. PC1 and PC2 represent the contribution rates of principal components. **B** The expression patterns of DEGs based on transcriptomic data. **C** Heatmap of the candidate DEGs related to calcium deficient apple fruit. T01, calcium deficiency apples; T02, healthy flesh of calcium deficiency apples; T03, healthy apple flesh

The PCA results showed that the contribution rates of PC1 and PC2 were 79.8% and 20.2%, respectively. In PC1, Ca and MDA contents were the main factors. In PC2, TA and TSS were the main factors (Fig. 5, Table S1). Correlation analysis indicated that Ca content showed a negative correlation with TSS (*person:* -0.345), sucrose (*person:* -0.4), •O_2_^−^ (*person:* -0.42) and MDA (*person:* -0.928), and it had a positive correlation with ascorbic acid (*person:* 0.576), glucose (*person:* 0.405), fructose (*person:* 0.709), H_2_O_2_ (*person:* 0.386), and TA (*person:* 0.719). In addition, TSS had a positive correlation with sucrose (*person:* 0.713); TA had a negative correlation with TSS (*person:* -0.239) and sucrose (*person:* -0.125) (Fig. S1, Table. S2).

### Identification of the candidate genes related calcium deficiency metabolism

According to transcriptome data of calcium deficiency “Fuji” apple fruit (T01), calcium deficiency apple healthy flesh (T02) and healthy apple fruit (T03) at fruit ripening period [7], eight expression patterns of all differentially expressed genes (DEGs) were obtained. We selected a total of 42 DEGs from profiles 0, 1, 6 and 7 (Fig. 5B). Among of them, 24 WRKY transcription factors, eleven genes of sugar metabolism (SS, SSL, SWEET and SPS), five genes of apoptosis and two genes of calcium signal have the same or opposite expression trends (Fig. 5C). Furthermore, promoters of genes encoding sucrose synthesis and transport enzymes, specifically *MdSWEET1-like* (MD10G101220)*, MdSWEET2a-like*(MD10G1269300)*, MdSWEET15* (MD16G1125300), *MdSPS2* (MD04G1013500)*, MdSS2* (MD02G1100600)*, MdSPS4* (MD05G1006400)*, MdSS* (MD15G1223500)*, MdSSL* (MD02G1100500) and *MdSWEET1* (MD10G1012200), were predicted by PlantCare (http://bioinformatics.psb.ugent.be/webtools/plantcare/html/). We found that there were W-box cis-elements in their 2000 bp upstream promoters. This suggested that WRKY TFs may be involved in the regulation of fruit sugar accumulation by binding to genes encoding sucrose synthesis and transport enzymes.

### *MdWRKY75* was related to *MdSWEET1* by qRT-PCR and correlation analysis

**Fig. 6:**
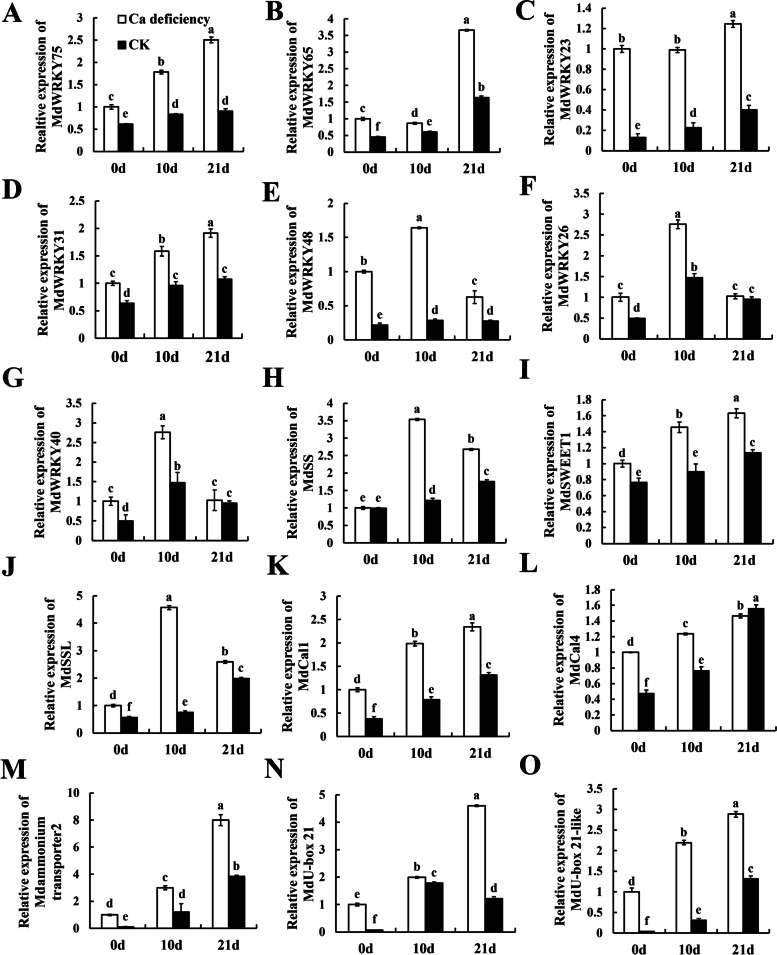
Evaluating the expression levels of candidate genes in postharvest healthy apples and calcium deficiency apples. **A** *MdWRKY75*, **B** *MdWRKY65*, **C** *MdWRKY23*, **D** *MdWRKY31*, **E** *MdWRKY48*, **F** *MdWRKY26*, **G** *MdWRKY40*, **H** *MdSS*, **I** *MdSWEET1*, **J** *MdSSL*, **K** *MdCal1, L* *MdCal4*, **M** *MdAmmonium transporter2*, **N** *MdU-box21* and **O** *MdU-box21-like.* Data are presented as means ± SD (*n* = 3). Different lower-case letters above the columns stand for significant difference between two values (*p* < 0.05) at the same time point

In order to further confirm the expression pattern of the above candidate genes in calcium deficiency apple fruit and healthy apple fruit at 0, 14, and 21 DAS, we determined the expression levels of *MdWRKY75* (MD13G1122100), *MdWRKY65* (MD05G1295700), *MdWRKY23* (MD17G1278100), *MdWRKY31* (MD05G1349800), *MdWRKY48* (MD13G1150700), *MdWRKY26* (MD03G1057400), *MdWRKY40* (MD00G1143500), *MdSSL* (MD02G1100500), *MdSS* (MD15G1223500), *MdSWEET1* (MD10G1012200) *MdAmmonium transporter* (MD12G1174700), *MdU-box 21* (MD13G1017300) and *MdU-box 21-like* (MD16G1015400) by qRT-PCR. As shown in Fig. 6, the expression levels of *MdSS, MdSSL, MdSWEET1, MdAmmonium transporter*, *MdU-box 21* and *MdU-box 21-like* were higher in calcium deficiency apple fruit than those of the CK fruit (*p* < *0.05*). The expression levels of WRKYs in calcium deficiency apple fruit were always higher than those in the CK fruit. In particular, the expression patterns of *MdWRKY75* and *MdWRKY31* were similar to those of *MdSWEET1*.

**Fig. 7:**
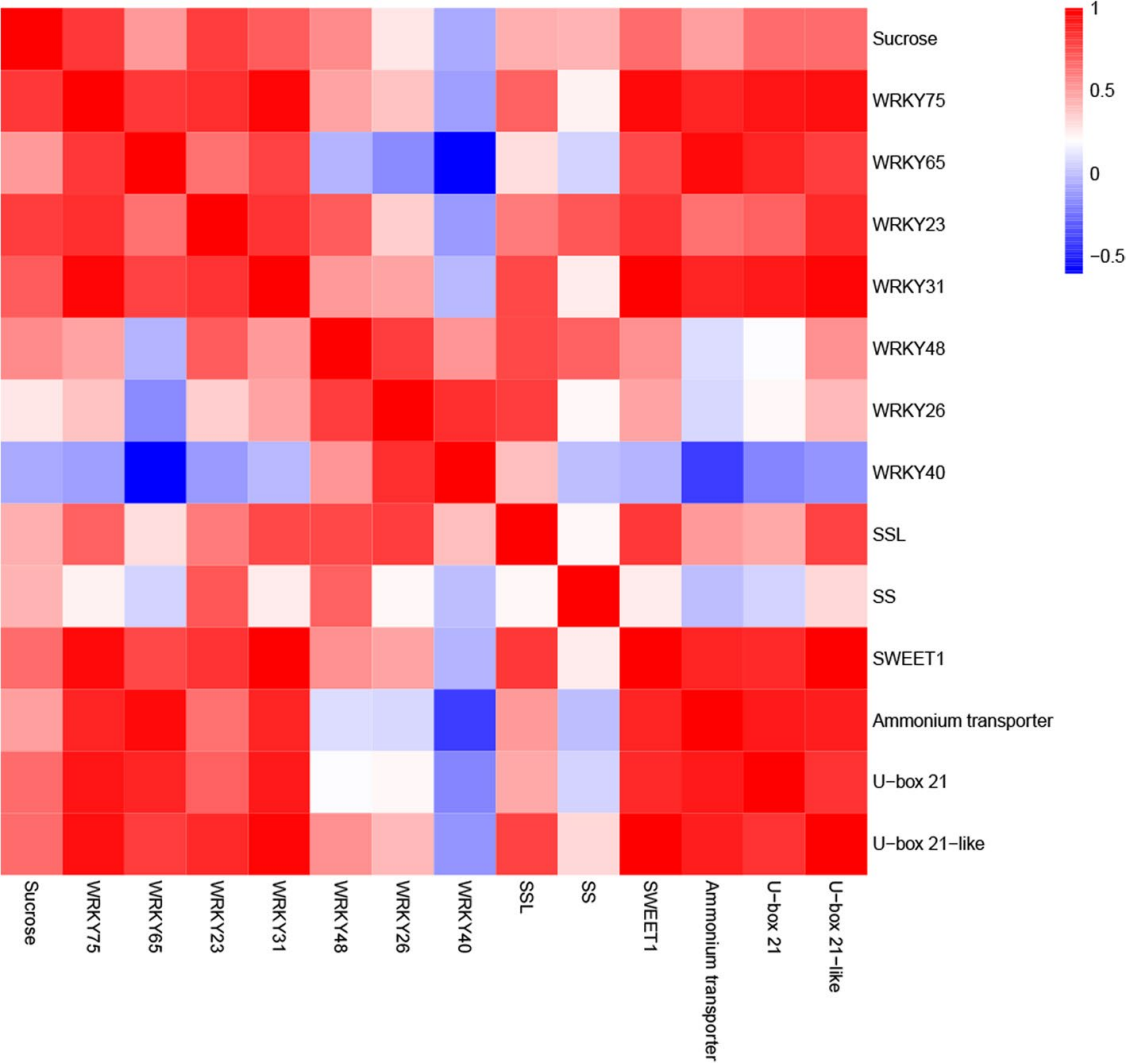
Correlation analysis of the relationship between sucrose content and the expression levels of candidate genes

Furthermore, correlation analysis showed that sucrose had positive correlation with *MdWRKY75* (*pearson*: 0.815), *MdWRKY23* (*pearson*: 0.802). Meanwhile, *MdWRKY75* (*pearson*: 0.959) and *MdWRKY31* (*pearson*: 0.987) had positive correlations with *MdSWEET1* (Fig. 7, Table. S3). Thus, sucrose accumulation was significantly positively correlated with *MdWRKY75* and *MdWRKY23,* the TFs *MdWRKY75* and *MdWRKY31* were related to *MdSWEET1*. Moreover, *MdWRKY75* had a positive correlation with *MdAmmonium transporter* (*pearson*: 0.88), *MdU-box 21* (*pearson*: 0.92) and *MdU-box 21-like* (*pearson*: 0.95). These results indicated that *MdWRKY75* might be regulate the expression of *MdSWEET1* and result in accelerating sucrose accumulation, and might be related to apoptosis in calcium deficiency apple fruit.

### Transient transformation of *MdWRKY75* in apple fruit

**Fig. 8:**
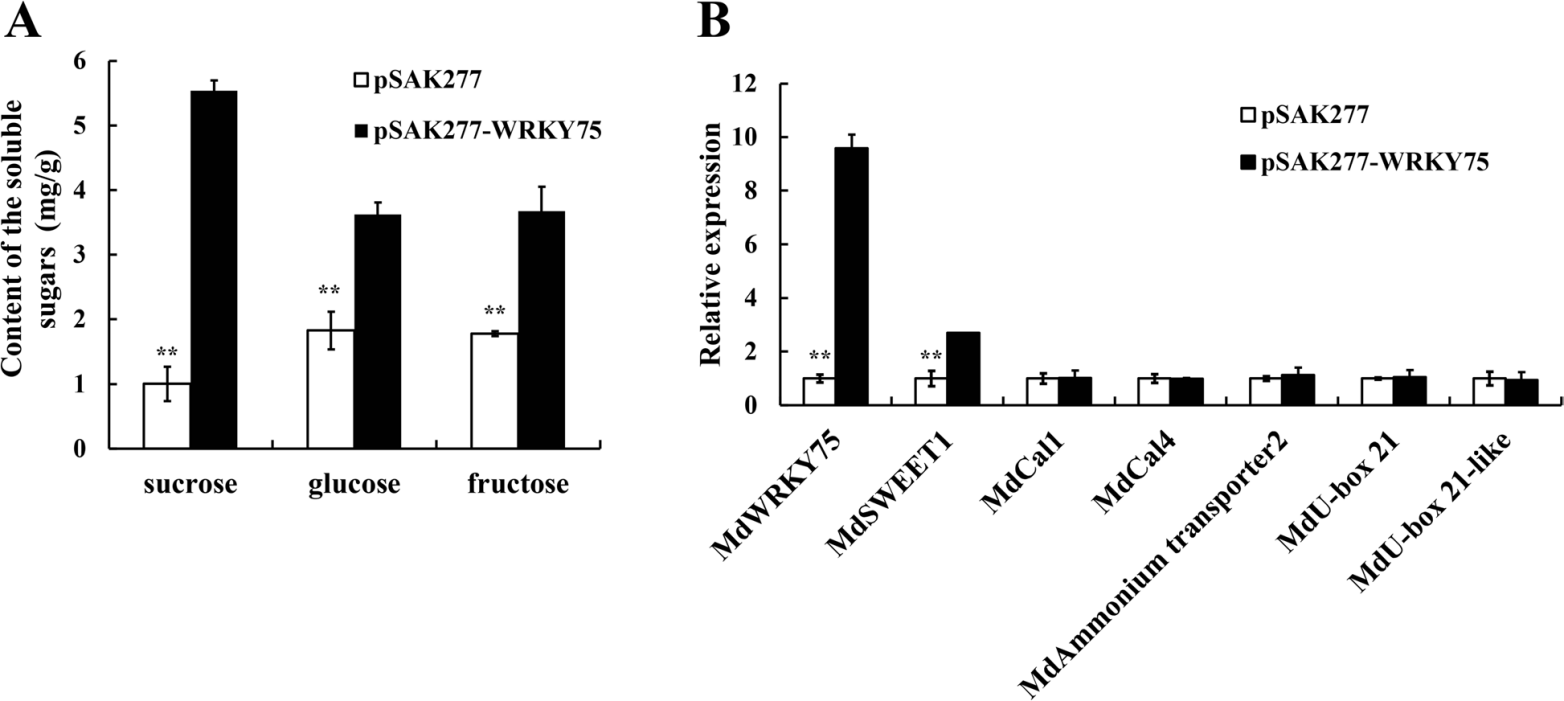
The changes of sucrose, glucose, fructose and related genes expression levels in apples infected pSAK277-*MdWRKY75*. **A** Changes in sucrose, glucose and fructose in apple fruit between empty vector and pSAK277-*MdWRKY75*. **B** Evaluating the expression levels of *MdWRKY75*, *MdSWEET1*, *MdCal1*, *MdCal4*, *MdAmmonium transporter2*, *MdU-box 21* and *MdU-box 21-like* in apple fruit between infected by empty vector and pSAK277-*MdWRKY75*. Data are presented as means ± SD (*n* = 3). * and ** indicate significance at *p* < 0.01 and* p* < 0.05, respectively

Because of *MdWRKY75* have a high expression level in calcium deficient apples, and have positive correlation with sucrose content and the expression of apoptosis related genes, we injected *MdWRKY75* into apple fruit and measured the sugar content and the expression level of sugar-, Ca- and apoptosis- related genes. As shown in Fig. 8A, the content of sucrose, glucose and fructose in apple fruit were higher than those of the empty vector (pSAK277). Especially, the sucrose content of *MdWRKY75-*oe in apple fruit is five fold higher than those of the empty vector(*p *< *0.01*). qRT-PCR analysis also showed that the expression levels of *MdWRKY75* and *MdSWEET1* were higher in *MdWRKY75* induced apple fruit than those of the empty vector (*p *< *0.01*)(Fig. 8B). However, *MdCal1*, *MdCal4*, *MdAmmonium transporter*, *MdU-box 21* and *MdU-box 21-like* do not change obviously (Fig. 8B).

## Discussion

### Calcium deficiency apples significantly stimulated the activity of ROS and decreased antioxidant capacity

MDA is one of the mainly products of membrane lipid peroxidation and can be used to reflect the degree of membrane peroxidation. It could increase with calcium deficiency and change the configuration of protein nucleic acid and ingrain cross-linking reaction thereby to lead to biological function lost eventually [29]. ·O_2_^−^ could derive into H_2_O_2_ and hydroxyl free radicals (OH^−^) in the organism, leading to lipid peroxidation damage of cell membranes thereby aging and disintegration. Generally, high activity degree of PPO also represented the deterioration level of fruit in postharvest because of its redox function which oxidizes phenolic substances into quinone compounds, lead the flesh of the fruit become brown [30]. In this study, the levels of ·O_2_^−^, MDA and PPO activity in calcium deficiency apples were higher compared with control apples (except H_2_O_2_) (Fig. 2). Our results are accordance with the changes of fruit senescence indexes [29 ~ 30], and it is similar to a report on which sufficient calcium can reduce the degradation of ascorbic acid and enhance the total antioxidant capacity in sweet cherry and ‘Royal delicious’ apple [14, 17]. Thus, the antioxidant capacity of ‘Honeycrisp’ apple with calcium deficiency decreased, ROS metabolism excessively accumulated, result in apple senescence appearance.

### The deterioration of calcium deficiency apples was faster than that of healthy apples

In our study, calcium deficiency can accelerate the senescence of apples during storage. There were also significant differences of the nutritional metabolites, such as flavonoids, total phenols, TSS and TA between calcium deficiency apples and healthy apples during postharvest storage, which strongly suggested that calcium played a key role in apple senescence. The contents of flavonoids and total phenols in calcium deficiency apples were higher than those in healthy apples (Fig. 3A, B), it suggested that calcium deficiency made apples show a higher level of maturity compared with healthy apples in the same period. Furthermore, TSS and TSS/TA increased, TA and ascorbic acid decreased in calcium deficiency apples (Fig. 4), it is showed that the taste of calcium deficiency apples is sweeter. Meanwhile, the sucrose content of calcium deficiency apple fruit was higher than that of healthy apples (Fig. 4E), providing direct evidence that calcium deficiency accelerates the accumulation of sweet substances. After fruits harvested, all the energy and intermediates required for its life activities come from the oxidation and decomposition process of sugars, so the level of sugar content reflects the quality and storage performance of the fruits. And the change of TA and carbohydrate contents of banana without exogenous calcium treatment were consistent with our results [14]. Additionally, the PCA and correlation analysis showed that the main factor Ca was positively correlated with TA and negatively correlated with TSS accumulation, TSS was positively correlated with sucrose accumulation (*pearson*: 0.71) (Fig. 5A, Table. S2). The results showed that Ca content was positively correlated with the accumulation of acidic substances and negatively correlated with the accumulation of sweet substances, thereby to indicated that deterioration of apples is related to calcium deficiency in apples.

### *MdWRKY75-MdSWEET1* is a potential regulatory model of sucrose transport in calcium deficiency apples

Sugar content was an important criterion for evaluating fruit maturity. The sugar/acid ratio is an index that affects fruit nutritional quality. In this study, we found that sucrose content and the related genes of sugar metabolism (*MdSS, MdSSL, MdSWEET1, MdWRKYs*) of calcium deficiency apples was higher than those of healthy apples (Fig. 5C). Meanwhile, *MdSS* and *MdSWEET1* had a positive correlation with TSS content and a negative correlation with Ca content (Fig. 7, Table S3), and *MdWRKY75* had a strongly positive correlation with *MdSWEET1* by correlation analysis. It suggested that *MdSWEET1* might be regulated by *MdWRKY75* thereby to affect sugar accumulation. Thus, we overexpressed *MdWRKY75* in apple fruit by injecting pSAK277-*MdWRKY75,* resulting in sucrose content and expression level of *MdSWEET1* increased (Fig. 8). This suggests that *MdWRKY75* can activate the expression of *MdSWEET1* to increase the accumulation of sucrose in calcium deficient apple. It was similar regulatory mechanism with sugar accumulation in pear fruit, it was reported that *PuWRKY31* can bind to the promoter of *PuSWEET15* to regulate sugar accumulation [28]. In addition, *MdAmmonium transporter*, *MdU-box 21* and *MdU-box 21-like* were expressed in calcium deficiency apples. This suggested calcium deficiency also activated apoptosis. Furthermore, *MdAmmonium transporter*, *MdU-box 21*, *MdU-box 21-like*, *MdCal1* and *MdCal4* have no obvious change in apple with over-expressing *MdWRKY75* (Fig. 8B). It implied that *MdWRKY75* cannot regulate the expression of calcium signaling and apoptosis related genes in apple, but apoptosis related genes may be involved in the sucrose metabolic pathway of apple fruit and affect the accumulation of sucrose. This study further strengthened the regulatory mechanism of calcium deficiency apple flesh and contributed to improving the appearance quality of apple fruit.

## Conclusions

**Fig. 9:**
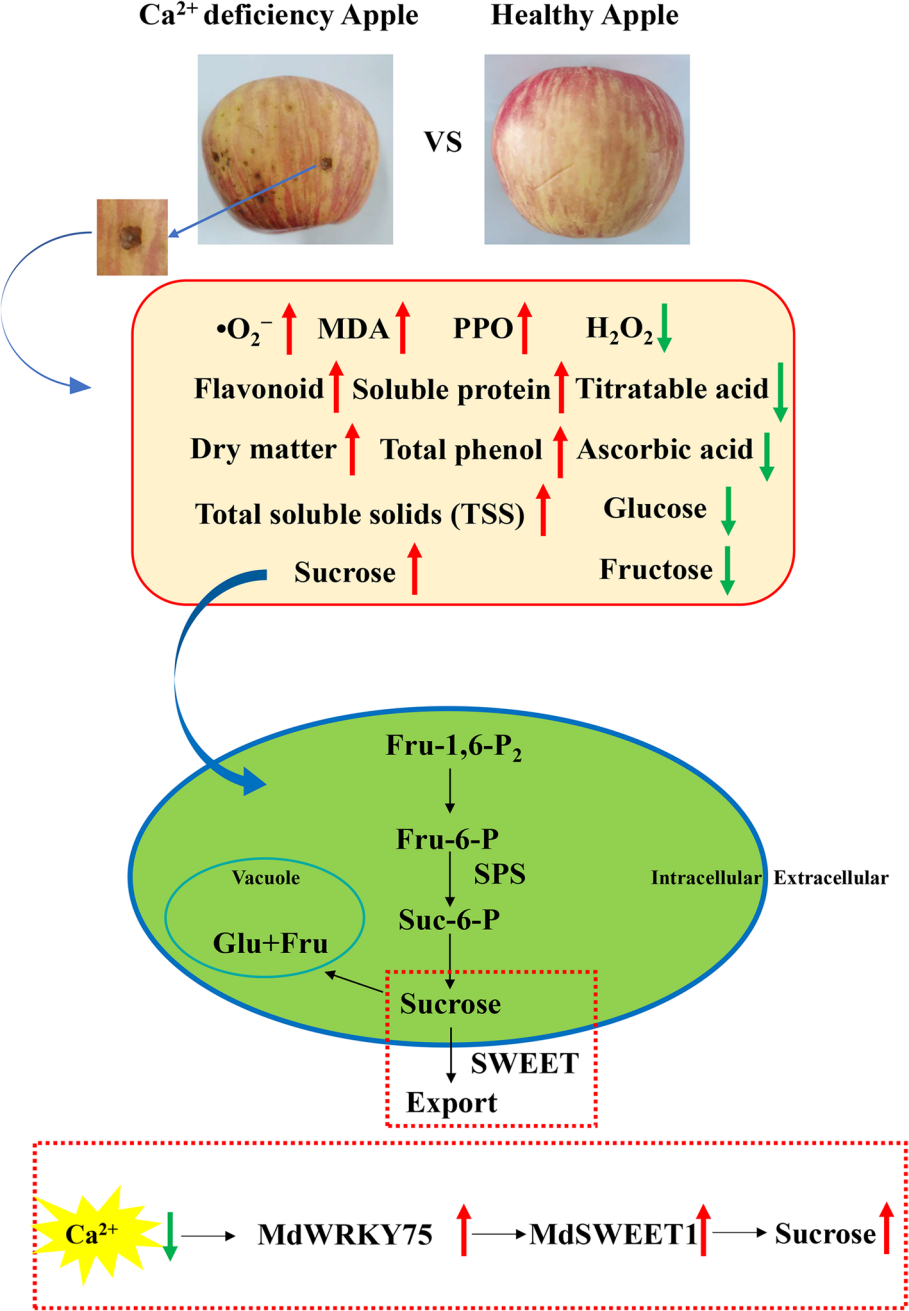
A proposed model of the mechanism of sucrose accumulation, which in apples with Ca deficiency. The red arrow represented the up-regulated expression. The green arrow represented down-regulated expression

In summary, this study found that the deterioration of calcium deficiency apples, including nutrients and antioxidant capacity, was faster than that of healthy apples. The results also indicated that the TSS and sucrose contents of calcium deficiency apples were higher than those of CK during storage. TSS, sucrose, ROS and Ca were identified as the main factors by PCA. In addition, transcriptome data mining, qRT-PCR analysis and transient expression indicated that *MdWRKY75* could activate the expression of sucrose metabolism-related enzyme *MdSWEET1* in Ca-deficient apple fruit. It suggested that *MdWRKY75* could bind to the *MdSWEET1* promoter by W-box cis-elements, and then promotes the contents of sucrose, glucose and fructose in apple fruit. Based on the results of our research, a model is proposed to develop a significant understanding of Ca^2+^ deficiency affecting TSS content through the sucrose metabolic pathway in apples with bitter pit (Fig. 9). Thus, this study provided a deep basis for sugar accumulation in fruit based on sucrose accumulation in calcium deficiency apples and improved fruit quality.

## Methods

### Plant materials and treatment

The healthy and calcium deficiency ‘Honeycrisp’ apple fruit used in this study were provided by Shandong Academy of Agricultural Sciences (Tai’an, Shandong Province) in 2018. During the ripening period, 60 calcium deficient apple fruits were harvested from the trees with the poor management level and the poor soils, and 60 healthy fruits without pests or mechanical damage were harvested from the trees with the normally management level as the controls (CK). Every ten healthy fruits/ calcium deficient were randomly divided into one group, and they were respectively placed in six 350 mm glass-vacuum-dryer with the appropriate amount of distilled water to maintain 75 ~ 85% relative humidity at room temperature (25 ℃). Ten apple fruits (without seeds and skins) were sampled every seven days until 21 days after storage (DAS). The samples were frozen in liquid N_2_ quickly and stored in a -80 °C refrigerator for subsequent experiments.

### Determination of Ca content in apple flesh

The Ca content of apple flesh was determined by flame atomic absorption spectrometry (FAAS) according to the methods described by Barea-Álvarez et al. [31]. Apple flesh (1 g, dry weight) was carbonized on a crucible. Then, samples were transferred to a high-temperature muffle furnace, and the temperature gradient was raised to 500 ℃ (50 ℃/30 min) until samples were burnt to white gray. When the samples cooled, they were mixed with 15 mL of a mixture of HNO_3_ and HClO_4_ (5:1 v/v). The best parameters for determination were *λ* = 422.7, current = 10 mA*,* and spectral resolution = 1.2 nm, and the gases were C_2_H_2_ and air (C_2_H_2_ 3.0 L·min^−1^; atmospheric air 13 L·min^−1^) (Hitachi Z2000). CaCl_2_ solution was used as the standard for calibration.

### Determination of flavonoid and total phenol contents and polyphenol oxidase activity

The flavonoid contents were assayed as described by Li et al. [32], and the absorbance was determined at 510 nm. Rutin was used as the standard for calibration.

Total phenols were assayed following the method of Pirie and Mullins [33]. The total phenols were determined by spectrophotometry at 280 nm. Gallic acid was used as the standard to make a calibration curve.

The activity of polyphenol oxidase (PPO) was determined by procedures described by Benjamin and Montgomery [34]. Apple flesh (5.0 ± 0.01 g) was ground in 5 ml of ice-cold extraction buffer [1 mM PEG, 4% polyvinylpyrrolidone (PPVP), 1% Triton X-100]. The homogenate was centrifuged, then the supernatant was collected and add to enzymatic substrate buffer [50 mM Catecholacetic, pH 5.5, 0.1 M acid-sodium acetate buffer] for protease activity determination. Absorbance was recorded at 400 nm, and the protease activity was quantified as U/g FW.

### Determination of malondialdehyde (MDA), hydrogen peroxide (H_2_O_2_), and superoxide anion (•O_2_^−^)

MDA, H_2_O_2_, and •O_2_^−^ were determined according to the methods described by Hu et al. [35]. Absorbance was recorded at 532 nm, and the value for nonspecific absorption at 600 nm was subtracted to obtain the MDA content. Values are expressed as μmol/g.

The H_2_O_2_ content was obtained by determining the absorbance at 508 nm and is indicated as micromoles per gram.

Superoxide (• O_2_^−^) production was calculated with an extinction coefficient of 2.16 × 104 M^−1^·cm^−1^. Corrections were made for the background absorbance in the presence of 50 units of superoxide dismutase (SOD) and presented as nmol/min/g.

### Detection of soluble protein and dry matter contents

For soluble protein, the supernatant was mixed with coomassie brilliant blue, and the absorbance was determined at 595 nm after 5 min according to the method described by Bradford [36]. The results are expressed as milligrams per gram of FW (fresh weight).

For dry matter, samples (5.00 ± 0.05 g) were placed in an oven at 80 °C until the weight at the third weighing remained unchanged. Then, the proportion relative to the initial weight was calculated.

### Determination of total soluble solid contents, ascorbic acid and titratable acids

Total soluble solids (TSS) were determined in samples of apple fruit (5.00 ± 0.05 g). After grinding and centrifugation (4000 r/min, 10 min), the juice was measured by an Abbe Refractometer (JH-WYA2S, Jiahang Instrument Co., Ltd, Shanghai, China). The titratable acid (TA) content was determined by acid–base neutralization with NaOH [37]. TSS and TA contents were presented as mg/g. The extraction and determination of ascorbic acid in apples followed the method of Nath et al. [38], and the assessment was performed by indophenol titration with minor changes, with values expressed as mg/100 g.

### Measurements of the soluble sugar content in apple fleshes

The soluble sugars in apple flesh were extracted following the method of Li et al. [28]. Briefly, two grams of the fruit flesh was homogenized, mixed with 5 ml of sterile deionized water, incubated in a water bath at 80 ℃ for 30 min, and then extracted by ultrasonic for 30 min at 50 W. Finally, the supernatant was collected by centrifugation (12,000 × g, 5 min) and filtration through a 0.22 μm membrane. The soluble sugars were measured by HPLC (Agilent Technologies 1260 Series) following the method of Jia et al. [39]. HPLC (Agilent 1260) was performed with a 7.8 × 300 mm Carbomix Ca-NP column (Sepax); the mobile phase was ultrapure water with a flow rate of 1 ml min^−1^; the column temperature at 80 °C; the refractive index detector temperature at 35 °C, and the injection volume was 20 μl. At each sampling point, at least five fruits were randomly selected and divided into three groups as three biological replicates.

### Quantitative real time PCR analysis

Total RNA from 0.1 g frozen apple fruit samples was extracted by an RNA Extraction Kit (Tiangen, Beijing, China). Then, cDNA was synthesized by a reverse transcription kit (PrimeScript RT Master Mix, Takara, Kyoto, Japan) and further used for quantitative PCR. The specific primers used for qPCR are listed in Supplementary Table S4. *MdTUB* (*TUB*, accession number GO562615) and *MdUBQ* (*UBQ*, accession number MDU74358) were used as housekeeping genes for the normalization of data. All data are expressed as the means and standard deviations of the values obtained from three biological replicates.

### Gene cloning and transient transformation of *MdWRKY75* in apple fruit

The full coding DNA sequence (CDS) of *MdWRKY75* (MD13G1122100) in apple was obtained by GDR (https://www.rosaceae.org/), and PCR amplification was conducted using Phanta Super-Fidelity DNA Polymerase (P501-d1, Vazyme Biotech Co. Ltd., China) and the primer sequences listed in Table S4. The full CDS fragment of *WRKY75* was inserted into pSAK277 vector under the control of the 35S promoter with *EcoR*I and *Xho*I. The recombinant expression vector WRKY75-pSAK277 was transformed into Agrobacterium tumefaciens (GV3101), it was cultured at 37 °C, and then collected, subsequently resuspended in a solution (included 10 mm MES, 10 mm MgCl_2_, and 200 μm acetosyringone) to a final optical density of 0.8 ~ 1.0 at OD_600_, and then incubated at room temperature for 3 − 4 h. The infiltration protocol and culture methods for transient expression were adapted from previously described methods [40, 41]. The infected apples were placed at 23 °C for 3 days. All fruit samples were frozen in liquid nitrogen upon collection, and stored at − 80 °C.

### Statistical Analysis

Statistical analysis was performed using a *t*-test and one-way ANOVA in SPSS 22.0. PCA was performed using factor analysis in dimension reduction, and the rotation method was carried out by varimax with Kaiser normalization. Correlation analysis and heatmap analysis were performed by R studio software.

## Supplementary Information

**Additional file 1.**

## Data Availability

All data generated and analyzed during this study are included in this published article. The databases used in this study as follows, GDR: https://www.rosaceae.org/ (open) NCBI database: https://www.ncbi.nlm.nih.gov/bioproject/PRJNA733599 (open)

## Abbreviations

Ca: Calcium
DAS: Days After Storage
•O_2_^−^: Superoxide Anion
MDA: Malondialdehyde
H_2_O_2_: Hydrogen Peroxide
PPO: Polyphenol Oxidase
PCA: Principal Component Analysis
DEG: Differentially Expressed Gene
TA: Titratable Acids
TSS: Total Soluble Solids
qRT-PCR: Quantitative Real Time PCR
FAAS: Flame Atomic Absorption Spectrometry
SS: Sucrose Synthase
SWEET: Sugars Will Eventually be Exported Ttransporters
SPS: Sucrose-Phosphate Synthase
ROS: Reactive Oxygen Species
