# Supplementary material for: Comparative physiological and transcriptomic analysis reveal MdWRKY75 associated with sucrose accumulation in postharvest ‘Honeycrisp’ apples with bitter pit
Source: BMC Plant Biol. 2022 Feb 17;22:71. doi: 10.1186/s12870-022-03453-8 (PMC8851858; doi:10.1186/s12870-022-03453-8)
Supplement: Supplementary file 1 — Additional file 1. [file 12870_2022_3453_MOESM1_ESM.docx]

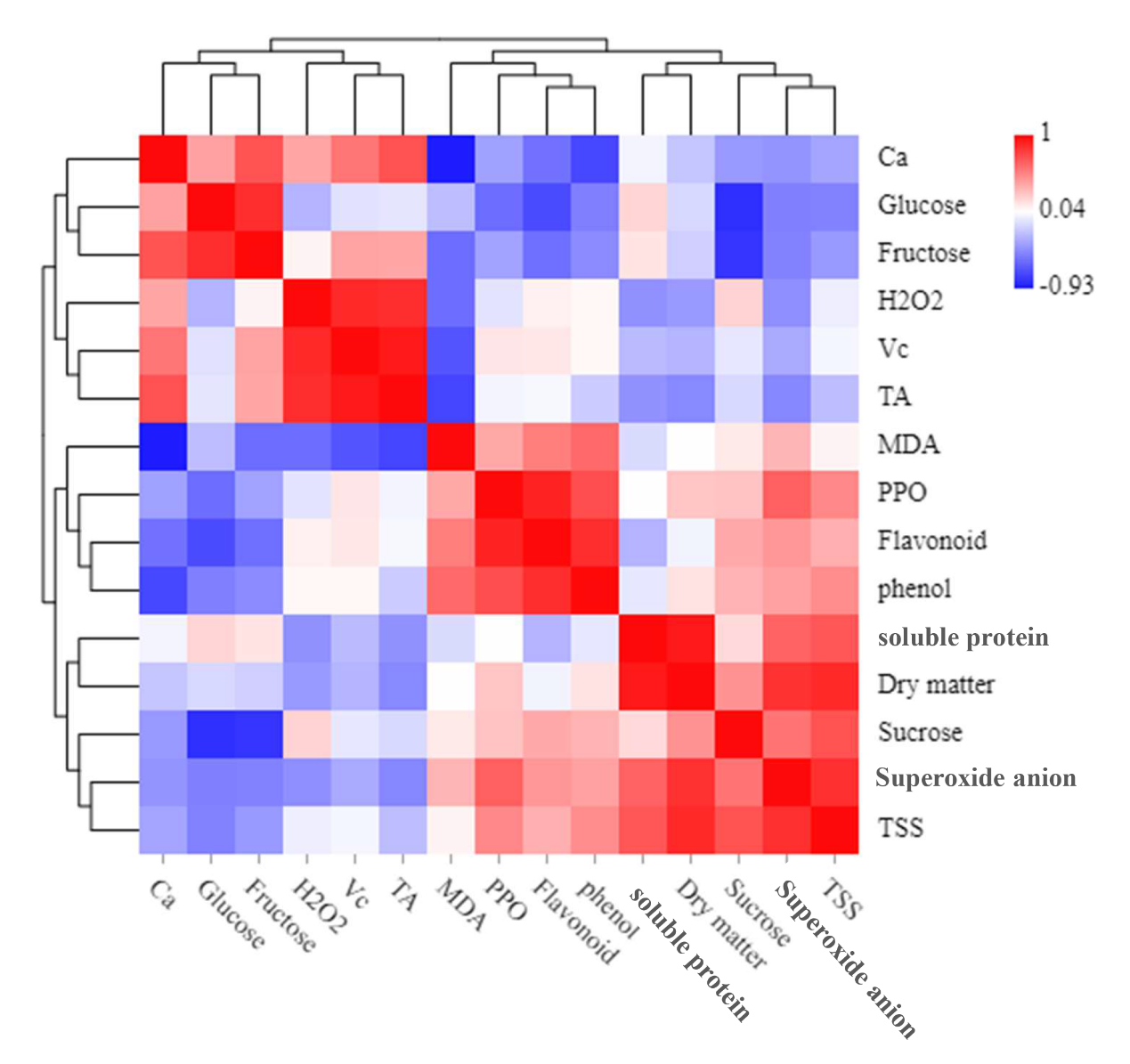


**Figure S1.** The correlation analysis the relationship among bioactive compounds. Ascorbic acid, Vc; Titratable Acids, TA; Malondialdehyde, MDA; Polyphenol Oxidase, PPO; Total Soluble Solids, TSS.

**Table S1.** The factors score of all the metabolites by principal component analysis in apple.

| **Component name** | **PC1 (79.8%)** | **PC2 (20.2%)** |
| --- | --- | --- |
| MDA content | -0.2904 | -0.21035 |
| PPO activity | -0.28007 | 0.258093 |
| Total soluble solids content | -0.18614 | 0.196467 |
| Ascorbate content | 0.130971 | 0.453404 |
| ·O_2_^-^ production rate | -0.32491 | 0.018726 |
| H_2_O_2_ content | 0.222245 | 0.360317 |
| Titratable acid content | 0.197565 | 0.416948 |
| Soluble protein content | -0.1777 | -0.02264 |
| Calcium content | 0.32276 | 0.118155 |
| Dry matter content | -0.30333 | 0.209257 |
| Total phenol content | -0.30967 | 0.13092 |
| Flavonoid content | -0.30333 | 0.209257 |
| Sucrose content | -0.09148 | 0.447216 |
| Glucose content | 0.297909 | -0.10415 |
| Fructose content | 0.280449 | 0.104416 |

**Table S2.** The correlation coefficient of correlation analysis among bioactive compounds.

| var | Ca | MDA | O2 | H_2_O_2_ | TSS | Vc | TA | Suc | Glu | Fru | PPO | SP | Flavonoid | phenol | Dry matter |
| --- | --- | --- | --- | --- | --- | --- | --- | --- | --- | --- | --- | --- | --- | --- | --- |
| Ca | 1.00 |  |  |  |  |  |  |  |  |  |  |  |  |  |  |
| MDA | -0.93 | 1.00 |  |  |  |  |  |  |  |  |  |  |  |  |  |
| O2 | -0.42 | 0.33 | 1.00 |  |  |  |  |  |  |  |  |  |  |  |  |
| H_2_O_2_ | 0.39 | -0.58 | -0.44 | 1.00 |  |  |  |  |  |  |  |  |  |  |  |
| TSS | -0.35 | 0.08 | 0.85 | -0.03 | 1.00 |  |  |  |  |  |  |  |  |  |  |
| Vc | 0.58 | -0.69 | -0.33 | 0.88 | 0.00 | 1.00 |  |  |  |  |  |  |  |  |  |
| TA | 0.72 | -0.76 | -0.47 | 0.86 | -0.24 | 0.94 | 1.00 |  |  |  |  |  |  |  |  |
| Suc | -0.40 | 0.12 | 0.58 | 0.21 | 0.71 | -0.06 | -0.12 | 1.00 |  |  |  |  |  |  |  |
| Glu | 0.41 | -0.25 | -0.50 | -0.28 | -0.50 | -0.09 | -0.07 | -0.85 | 1.00 |  |  |  |  |  |  |
| Fru | 0.71 | -0.59 | -0.49 | 0.08 | -0.40 | 0.39 | 0.39 | -0.82 | 0.86 | 1.00 |  |  |  |  |  |
| PPO | -0.37 | 0.38 | 0.66 | -0.08 | 0.50 | 0.14 | -0.01 | 0.27 | -0.58 | -0.36 | 1.00 |  |  |  |  |
| SP | -0.01 | -0.12 | 0.65 | -0.43 | 0.70 | -0.26 | -0.43 | 0.18 | 0.20 | 0.14 | 0.05 | 1.00 |  |  |  |
| Flavonoid | -0.57 | 0.54 | 0.44 | 0.09 | 0.35 | 0.13 | 0.00 | 0.37 | -0.73 | -0.57 | 0.90 | -0.29 | 1.00 |  |  |
| phenol | -0.74 | 0.62 | 0.41 | 0.06 | 0.49 | 0.07 | -0.18 | 0.33 | -0.51 | -0.46 | 0.73 | -0.06 | 0.86 | 1.00 |  |
| Dry matter | -0.21 | 0.04 | 0.84 | -0.39 | 0.87 | -0.28 | -0.47 | 0.46 | -0.13 | -0.17 | 0.26 | 0.94 | -0.02 | 0.15 | 1.00 |

**Table S3.** The correlation coefficient of correlation analysis among and Sucrose contents and the genes of sucrose metabolizes.

| var | Sucrose | *WRKY75* | *WRKY65* | *WRKY23* | *WRKY31* | *WRKY48* | *WRKY26* | *WRKY40* | SSL | SS | SWEET1 |
| --- | --- | --- | --- | --- | --- | --- | --- | --- | --- | --- | --- |
| Sucrose | 1.00 |  |  |  |  |  |  |  |  |  |  |
| *WRKY75* | 0.81 | 1.00 |  |  |  |  |  |  |  |  |  |
| *WRKY65* | 0.52 | 0.81 | 1.00 |  |  |  |  |  |  |  |  |
| *WRKY23* | 0.80 | 0.84 | 0.64 | 1.00 |  |  |  |  |  |  |  |
| *WRKY31* | 0.71 | 0.98 | 0.78 | 0.83 | 1.00 |  |  |  |  |  |  |
| *WRKY48* | 0.56 | 0.48 | -0.03 | 0.70 | 0.52 | 1.00 |  |  |  |  |  |
| *WRKY26* | 0.26 | 0.38 | -0.16 | 0.36 | 0.48 | 0.80 | 1.00 |  |  |  |  |
| *WRKY40* | -0.06 | -0.10 | -0.60 | -0.12 | -0.01 | 0.53 | 0.85 | 1.00 |  |  |  |
| SSL | 0.44 | 0.69 | 0.30 | 0.61 | 0.77 | 0.77 | 0.81 | 0.40 | 1.00 |  |  |
| SS | 0.43 | 0.24 | 0.07 | 0.72 | 0.25 | 0.69 | 0.23 | 0.01 | 0.22 | 1.00 |  |
| SWEET1 | 0.66 | 0.96 | 0.77 | 0.83 | 0.99 | 0.54 | 0.48 | -0.04 | 0.82 | 0.25 | 1.00 |

**Table S4.** Real-time qPCR primers used in this study for DGEs.

| **Gene name** | **Gene ID** | **Prime sequence (5' to 3')** |
| --- | --- | --- |
| *MdWRKY75-F* | MD13G1122100 | CTTAGGGCTGATGTCAGAGATG |
| *MdWRKY75-R* |  | CACAGTTGCTTCACCACTTTC |
| *MdWRKY65-F* | MD05G1295700 | CTGAGCGATCAGGAAGAGAATG |
| *MdWRKY65-R* |  | GAGGTGGAAGTGGTGATCTTG |
| *MdWRKY23-F* | MD17G1278100 | TGAAGAAGGATGTGGTGAAGAG |
| *MdWRKY23-R* |  | CTGCAAATGGTGATGTGGTAAG |
| *MdWRKY31-F* | MD05G1349800 | CCCATGTCCTCGTGCATATT |
| *MdWRKY31-R* |  | GTGATCAGTATGGTTCTGTCCTC |
| *MdWRKY48-F* | MD13G1150700 | CGAACTCGTCTTCGATCTCTTC |
| *MdWRKY48-R* |  | TTTCTCCGGATCTTGCTCATC |
| *MdWRKY26-F* | MD03G1057400 | CTCAGACTTCTCCTTCCAAACC |
| *MdWRKY26-R* |  | CATGCCTGCTCTTGTGTAGT |
| *MdWRKY40-F* | MD00G1143500 | TAAGTCCCAATAGTGCCATGTG |
| *MdWRKY40-R* |  | GGATCTTTGGTCAGGGAAGAAG |
| *MdSSL-F* | MD02G1100500 | GGAGTCTATGGCTTCTGGAAAG |
| *MdSSL-R* |  | ACTTTCTCTTCACTCCACAGC |
| *MdSS-F* | MD15G1223500 | GAAGGACAGAAACAAGCCAATC |
| *MdSS-R* |  | TCACCAGCAACTACAACCAG |
| *MdSWEET1-F* | MD10G1012200 | TCCTTTGAGATTTGTGCGTTTG |
| *MdSWEET1-R* |  | CCCAAATGAAAGAATGCGAGAG |
| *MdCAL1-F* | MD14G1241000 | AAGTGCTTCATGAGTCTCGAC |
| *MdCAL1-R* |  | CCCAATTACATATCCAAACCACAG |
| *MdCAL4-F* | MD13G1151300 | CGAGGATCTGAAGAGCTACATG |
| *MdCAL4-R* |  | ATCAACGGCCAGGATCTTAAG |
| *MdAmmonium transporter-F* | MD12G1174700 | GCTAAACAAAGGCGACAACG |
| *MdAmmonium transporter-R* |  | CCATGAAGGCTGAGTTGACTG |
| *MdU-box21-F* | MD13G1017300 | GGGAGAGGTTGGAGTGTATTG |
| *MdU-box21-R* |  | AGTGGTTTGGTATGGTCAGC |
| *MdU-box21-like-F* | MD16G1015400 | TCACATGCCCTATTACCAACC |
| *MdU-box21-like-F* |  | TCTCGACGCACCAATCTTG |
| *MdWRKY75-pSAK277-F* | MD13G1122100 | actagtggatccaaagaattcATGGAAAATTACCCAACATTCTTTT |
| *MdWRKY75-pSAK277-R* |  | tcattaaagcaggactctagaGTAGCCTAGCTCGCAAAGAGCT |
